# Empirical Support for the Pattern of Competitive Exclusion between Insect Parasitic Fungi

**DOI:** 10.3390/jof7050385

**Published:** 2021-05-14

**Authors:** Shiqin Li, Wenjuan Yi, Siyi Chen, Chengshu Wang

**Affiliations:** 1 CAS Center for Excellence in Molecular Plant Sciences, Key Laboratory of Insect Developmental and Evolutionary Biology, Shanghai Institute of Plant Physiology and Ecology, Chinese Academy of Sciences, Shanghai 200032, China; lishq1@shanghaitech.edu.cn (S.L.); 18270821134@163.com (W.Y.); chensy3@shanghaitech.edu.cn (S.C.); 2 School of Life Science and Technology, ShanghaiTech University, Shanghai 201210, China; 3 CAS Center for Excellence in Biotic Interactions, University of Chinese Academy of Sciences, Beijing 100049, China

**Keywords:** fungal parasite, insect host, competition, mycosis, coexistence, ecological niches

## Abstract

Fungal entomopathogens are largely facultative parasites and play an important role in controlling the density of insect populations in nature. A few species of these fungi have been used for biocontrol of insect pests. The pattern of the entomopathogen competition for insect individuals is still elusive. Here, we report the empirical competition for hosts or niches between the inter- and intra-species of the entomopathogens *Metarhizium robertsii* and *Beauveria bassiana*. It was found that the synergistic effect of coinfection on virulence increase was not evident, and the insects were largely killed and mycosed by *M. robertsii* independent of its initial co-inoculation dosage and infection order. For example, >90% dead insects were mycosed by *M. robertsii* even after immersion in a spore suspension with a mixture ratio of 9:1 for *B. bassiana* versus *M. robertsii*. The results thus support the pattern of competitive exclusion between insect pathogenic fungi that occurred from outside to inside the insect hosts. Even being inferior to compete for insects, *B. bassiana* could outcompete *M. robertsii* during co-culturing in liquid medium. It was also found that the one-sided mycosis of insects occurred during coinfection with different genotypic strains of either fungi. However, parasexual recombination was evident to take place between the compatible strains after coinfection. The data of this study can help explain the phenomena of the exclusive mycosis of insect individuals, but co-occurrence of entomopathogens in the fields, and suggest that the synergistic effect is questionable regarding the mixed use of fungal parasites for insect pest control.

## 1. Introduction

Fungi are the second most species-rich eukaryotic group after the insects [1,2]. After long-term coevolution, different relationships have been formed between insects and fungi such as the pattern of the insect infection and mycosis by parasitic fungi. Similar to the distribution features of diverse insect species, fungal parasites are largely cosmopolitan and sympatrically distributed in different ecosystems [3]. For example, it has been reported that the propagules of the ascomycete *Metarhizium* and *Beauveria* species could reach up to 10^6^ CFUs (colony forming units) per gram of soil [4,5,6]. It is therefore conceivable that the multiple species or strain spores of fungal parasites can land on individual insect hosts to theoretically trigger co-infections. In reality, however, the insect cadavers collected from the fields are always mycosed by a single species/strain of fungal parasites [7,8]. On the other hand, coenzootic or coepizootic fungal insect diseases have been frequently observed in the field, where the same or different species of insects were killed and mycosed by different strains or species of fungal parasites [8,9,10]. Regarding the competition and coexistence of the multiple parasites of plants and mammals, different models have been proposed by theoretical ecology, such as the competitive exclusion [11,12], competition–colonization tradeoffs [13,14], and modern coexistence theory [15]. The model and fate-determining factors remain unclear regarding the competition for niches, including hosts, between insect pathogenic fungi.

Except for the elusive pattern of competition, different species of entomopathogenic fungi have been developed and used as environmentally-friendly mycoinsecticides for biocontrol of insect pests [16,17]. In particular, efforts were considered to co-formulate the spores of different strains or species of pathogenic fungi for better control of insect pests by overcoming environmental constraints such as irregular temperature fluctuations [18,19,20]. However, the synergistic effect was not evident or questioned [18]. The potential or degree of cross inhibition between entomopathogens was largely overlooked before co-formulations.

Field application and recovery studies revealed that the inundative application of mycoinsecticide agents such as *B. bassiana* did not displace local population. Instead, different genotypic strains coexist in the field by infection of different insects, and less than 3% of the insect cadavers were mycosed by the released strains [7,21]. These kinds of studies have not investigated the effect of biocontrol releases on other species of fungal parasites. Entomopathogenic fungi infect hosts through penetration of insect cuticles and then colonization of insect hemocoels by overcoming host immunities [22]. It is still unclear regarding the stage-wise competition and factors that may essentially determine the competitive coexistence of fungal insect parasites.

In this study, we sought to provide empirical insights into the outcomes and patterns of competition between fungal insect parasites. By coinfection of insects with different species and strains of *M. robertsii* and *B. bassiana* each with a wide host range, we report the dominant mycosis of insect individuals by one species or one strain of the parasites. The model of competitive exclusion is empirically suggested in dependent or independent associations with factors such as the insect host species, initial titer, infection order, and growth media.

## 2. Materials and Methods

### 2.1. Fungal Strains and Growth Assays

Parasitic fungal strains of *B. bassiana* ARSEF 2860 (mating type, MAT1-1; abbreviated as Bb), ARSEF 8028 (MAT1-2) [23], and *M. robertsii* ARSEF 23 (MAT1-1; abbreviated as Mr) and ARSEF 2575 (MAT1-1) [24] were used in this study. In addition, red or green fluorescent protein (RFP/GFP) labeled strains were either generated by controlling gene expression with a constitutive promoter (i.e., the *Tef* gene promoter used for gene expression in *Metarhizium* and *Gpd* gene promoter used in *Beauveria*) or obtained from previous studies [25,26], including 2860-RFP, 8028-GFP, 23-GFP, and 2575-RFP strains. Fungal cultures were maintained on potato dextrose agar (PDA; BD Difco, Sparks Glencoe, MD, USA) for two weeks for harvesting conidial spores for bioassays against different insects. For growth assays, fungal strains were inoculated either individually (2 μL of 1 × 10^6^ conidia/mL) on PDA plates (9 cm in diameter) or in pairs on the same plates (2 cm spacing). To test the co-culturing in liquid medium, fungi were grown in Sabouraud dextrose broth (SDB, BD Difco, Sparks Glencoe, MD, USA) in a rotary shaker (190 rpm) for different times.

### 2.2. Insect Bioassays

Different fungal species/strains and their spore mixtures were used for both topical infection and injection assays against the last instar larvae of the wax moth *Galleria mellonella*, the females of the fruit fly *Drosophila melanogaster*, and the last instar larvae of the mealworm *Tenebrio molitor*. The wax moth and mealworm larvae were ordered from the company Keyun (Jiyuan, China). The fruit flies were reared on a standard cornmeal–agar medium at 25 °C, and the two-day-old adults post eclosion were used for bioassays [27]. The conidial suspensions of each strain were adjusted in 0.01% Tween 20 to the concentration of 1 × 10^7^ conidia/mL for topical infection assays, and 1 × 10^6^ conidia/mL for injections. Spore mixtures were prepared by mixing the suspensions of two fungal species or strains at the volume ratios of 1:9, 1:1, and 9:1, respectively. For topical infections, insects were immersed in spore suspensions for 30 sec. To examine the potential priority effect of fungal infection, we conducted topical infection of insects by treatment with either Bb or Mr for 16 and 24 h first and then re-immersion of the insects in Mr or Bb spore suspensions. For injections, each insect was injected with 20 μL of spore suspension from the second proleg using a micro-injector (Burkard manufacturing, Rickmansworth, UK). The insects immersed in or injected with 0.01% Tween 20 were used as reference controls. Insect mortality was recorded every 12 h. The median lethal time (LT_50_) of insects was estimated for each treatment, and the difference of insect survivals between treatments was calculated by Kaplan–Meir analysis [28]. The GFP/RFP-labelled strains were used for the same treatments. The newly dead insects were bled to count the fluorescent fungal cells formed within insect body cavities using a fluorescent microscope (Nikon Eclipse Ni, Tokyo, Japan).

For mycosis assays, the dead insects were lined on the moisturized filter papers for two weeks, and the mycosis ratio of insects by either or both fungi was estimated. Each treatment had three replicates (20 insects per replicate) and the experiments were repeated at least twice. After infection with the GFP-RFP-labelled strains, the fluorescent conidia formed on mycosed insects were then counted. The differences between the treatments with different species or strains were compared using the two-tailed Student’s *t*-test.

### 2.3. Penetration Test

To determine whether there was a cross-inhibition effect on fungal penetration, we inoculated both individual and mixed fungal spores on cellophane membranes lined on a minimal medium agar (NaNO_3_, 6 g/L; KCl, 0.52 g/L; MgSO_4_ 7H_2_O, 0.52 g/L; KH_2_PO_4_, 0.25 g/L; agar, 15 g/L) [29]. The spore suspensions of Bb and Mr were prepared in 0.01% Tween 20 to a final concentration of 5 × 10^6^ conidia/mL, and a mixture was prepared at the volume ratio of 1:1. The spore suspensions (2 μL each) were then individually inoculated on cellophane for three days. The membranes were then carefully removed together with fungal cultures, and the plates were then kept for incubation for an additional seven days to determine the outgrowth or not of fungal cultures [30]. Each treatment had 15 plates, and the experiments were repeated three times.

### 2.4. Germination and Appressorium Formation Assays

Spore suspensions (20 μL each; 1 × 10^8^ conidia/mL) of Bb, Mr, and Bb:Mr (1:1) were transferred to petri dishes (9 cm in diameter) containing 2 mL of the minimum medium containing 1% glycerol [31]. After incubation for 18 h, germination of both fungi and appressorium formation of Mr were examined under microscopy [30]. There were three replicates for each treatment, and at least five to six microscopic fields were examined for estimation of the fungal germination and appressorium formation rates.

### 2.5. Liquid Incubation and Biomass Quantification

To determine the growth competition between Bb and Mr in artificial medium, we inoculated the spore suspension (1 mL each of 1 × 10^8^ conidia/mL) of Bb, Mr, and Bb:Mr (at the ratios of 1:9; 1:1, and 9:1) into flasks (250 mL) containing 100 mL SDB medium. Mycelia were harvested seven days post incubation in a rotary incubator at 25 °C and 190 rpm. The samples were washed thrice with sterile water, dried at 65 °C overnight and weighed. There were five replicates for each treatment, and the experiments were repeated twice. To determine Bb and Mr cell ratio in co-cultures, mycelia of the three mixtures (i.e., Bb:Mr at the ratios of 1:9, 1:1, and 9:1) were harvested three and seven days post inoculation, and the dried samples (0.1 g each) were used for DNA extractions with a Hi-DNAsecure plant kit (TIANGEN, Beijing, China). After quantification, 1 pg of the plasmid pDHt-Bar was added into each DNA sample as a reference for real-time quantitative PCR analysis. PCR primers were designed for the *Bar* gene (BarF: GGAGGTCAACAATGAATGCC; BarR: ATGTCCTCGTTCCTGTCTGC; product size, 171 bp), the LysM effector *Blys2* gene [25] for Bb (Blys2F: ACCACTCTCACTACCGCTGT; Blys2R: CCTTGCCTGAATGAAGAGTT; product size 160 bp), and the perilipin *Mpl1* gene [32] for Mr (Mpl1F: TGTACAACGACACTCGCAGC; Mpl1R: CGACAAAGGCGGTAGAGACA; product size, 156 bp). After reaction, the DNA ratio of either Bb or Mr in different mixtures was calculated by reference to the spike-in plasmid DNA. The experiments were repeated twice.

## 3. Results

### 3.1. Non-Synergistic Effect of Coinfection on Fungal Virulence

**Figure 1:**
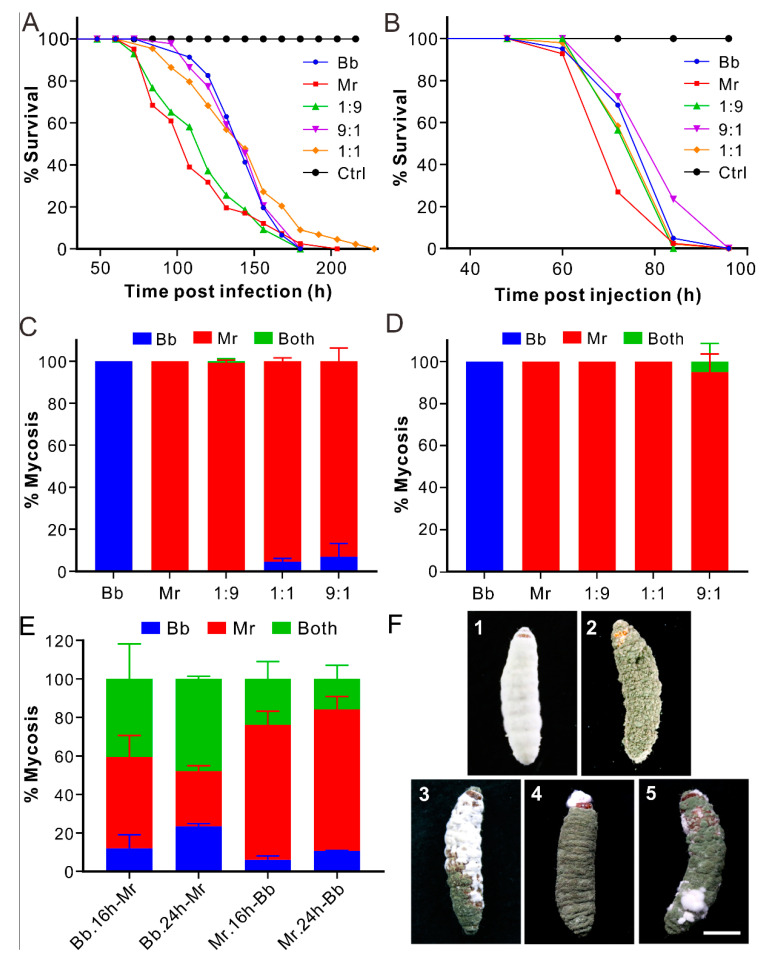
Insect survival and mycosis. (**A**) Survival of the wax moth larvae after topical infection. (**B**) Survival of the wax moth larvae after injection. (**C**) Percentage of the insect mycosis after topical infection of different parasites. (**D**) Percentage of the insect mycosis after injection of different parasites. (**E**) Percentage of the insect mycosis after sequential infection of different parasites. Strains: Bb, *Beauveria bassiana* ARSEF 2860; Mr, *Metarhizium robertsii* ARSEF 23. Treatments such as Bb.16h-Mr and Bb.24h-Mr mean that the insects were treated with the Bb spore suspension for 16 h or 24 h and then re-treated with the Mr suspension and vice versa. (**F**) Different type of mycosed insect cadavers. (**1**) A larva mycosed by Bb; (**2**) a larva mycosed by Mr; (**3**–**5**) larvae mycosed by both fungi in different types. Bar, 1 cm

To determine the virulence effect and mycosis fate of coinfection, we first used the *B. bassiana* strain ARSEF 2860 and *M. robertsii* strain ARSEF 23 for different experiments. These two strains had different growth rates on PDA; that Mr grew faster that Bb was evident after inoculation for 6 or 11 days (*t*-test: df = 4, *t* > 14, *p* < 0.001) (Figure S1A). After topical infection of the wax moth larvae, we found that the insects infected by Mr (with a median lethal time LT_50_ = 108 ± 4.2 h) died faster (log-rank test: df = 1, χ^2^ = 10.75, *p* = 0.001) than those treated with Bb (LT_50_ = 104 ± 4.0 h). The insect survival curves of those coinfections were largely in the middle of the dynamics caused by two individual strains (Figure 1A; Table S1). For injection assays, the survival trends of insects were rather similar to those treated by topical infections. The results showed that Mr (LT_50_ = 72 ± 1.3 h) killed insects faster (log-rank test: df = 1, χ^2^ = 11.12, *p* = 0.0009) than Bb (LT_50_ = 84 ± 0.64 h), and the survival of co-injection treatments had no significant difference with that of Bb injection but was in sharp contrast to that of Mr treatment (Figure 1B; Table S2).

For topical infection of either the mealworm larvae or fruit fly females, a non-synergistic effect of coinfections was similarly observed. Mr killed flies faster (log-rank test: df = 1, χ^2^ = 80.22, *p* < 0.0001) than Bb, and the 1:1 ratio coinfection curve was in the middle (Figure S2A; Table S3). For mealworm larvae, no obvious difference was observed (log-rank test: df = 2, χ^2^ = 3.21, *p* = 0.073) for insect survival after topical infection with the spore suspensions of Bb, Mr, and Bb/Mr mixture (1:1) (Figure S2B; Table S4). 

### 3.2. Dominant Mycosis of Insect Cadavers by Metarhizium

After estimation of insect mycosis ratios, unexpectedly we found that the wax moth cadavers were largely mycosed by Mr during both topical infection and injection assays. For example, with topical coinfection at the Bb:Mr ratio of 1:9, the cadavers were almost completely mycosed by Mr, and more than 90% of the cadavers were colonized by Mr at the Bb:Mr ratios of 1:1 and even 9:1 (Figure 1C). The mycosis rates were more highly biased towards Mr after the co-injections than topical coinfection assays. The former treatments resulted in the dominant occupation of insect cadavers by Mr at the Bb:Mr ratios of 1:9, 1:1, or 9:1 and a few cases of co-mycosis (i.e., the single cadaver mycosed by two fungi) at the 9:1 ratio (Figure 1D,F). The dead flies were also highly mycosed by Mr (>90%) after topical coinfection of Bb/Mr (1:1) (Figure S3A). For mealworms, however, the mycosis ratio of the coinfection treatment (1:1) varied in the increasing order of Bb (11.3 ± 1.5%), Mr (30.0 ± 3.0%), and co-mycosis (58.7 ± 2.3%) (Figure S3B). Thus, the species of insect hosts can affect the fate of mycosis after coinfection.

### 3.3. Biased Mycosis of Insect Cadavers after Sequential Infections

After the tests of priority effects, it was found that the Bb-first treatments did not result in the dominance of Bb mycosis; instead, the later treated Mr or co-mycosis took the bigger proportions (Figure 1E). For example, 24 h post immersion of Bb spores, further treatment with Mr resulted in 28.6 ± 2.8%of the Mr-mycosed cadavers and 47.9% ± 1.4 of co-mycosis by both Bb and Mr. However, the Mr-first treatments largely resulted in the Mr-mycosed cadavers, e.g., 70.1 ± 6.9%for the Mr inoculation for 16 h followed by the Bb treatment; and 73.5 ± 6.7% for the Mr inoculation for 24 h followed by the Bb treatment. The ratios were both significantly higher than those mycosed by Bb (*t*-test: df =2, *t* > 12, *p* < 0.01) (Figure 1E). Thus, the priority effect was not applicable to Bb. The phenotypes of insect mycosis are shown in Figure 1F. It is noteworthy that Mr usually took the bigger part of cadavers than Bb did during co-mycosis.

### 3.4. Dual Inhibition of Spore Germination or Appressorium Formation

**Figure 2:**
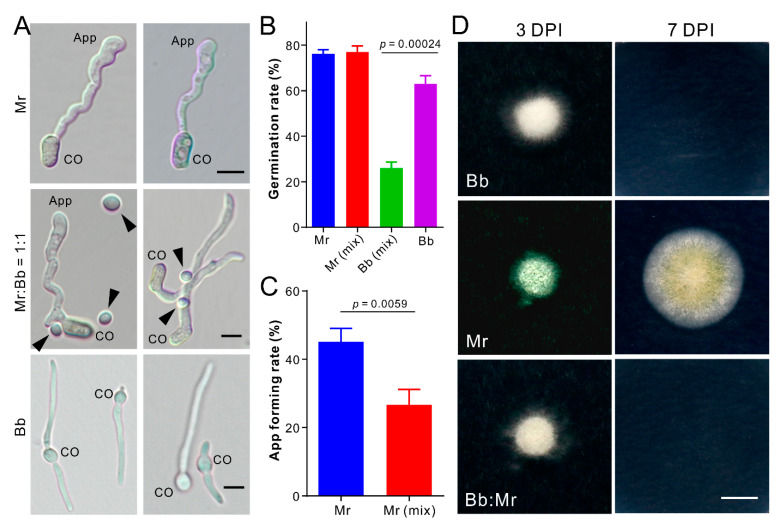
Dual inhibitions on spore germination, appressorium formation, and fungal penetration. (**A**) Microscopic imaging of spore germination and appressorium formation. Strains: Bb, *Beauveria bassiana* ARSEF 2860; Mr, *Metarhizium robertsii* ARSEF 23. CO, conidium; App, appressorium. Un-germinated Bb spores were arrowed in the mixing treatment (i.e., Bb:Mr =1:1). Bar, 5 µm. (**B**) Statistic of spore germination. Mr (mix) or Bb (mix) means the germination ratio of Mr or Bb spores in the mixture (1:1) treatment. (**C**) Comparative estimation of the Mr appressorium formation in pure and mixed cultures. (**D**) Penetration assays of Bb, Mr, and Bb:Mr (1:1) against the cellophane membrane. The membranes were carefully removed with fungal cultures three days post inoculation (DPI) and kept for incubation for additional seven days. Bar, 1 cm.

After examining the effect of conidial mixture on spore germination and induction of appressorium formation, it was found that the germination rate of the Mr spores in mixture (1:1) had no difference to that of the solo Mr spores. However, Bb spore germination was significantly (*t*-test: df = 4, *t =* 14.33, *p* = 0.0001) reduced in mixture when compared with its germination alone in medium (Figure 2A,B). In contrast to Bb, Mr spores germinate and produce the infection structure appressoria on hydrophobic surface. The appressorium formation rate of Mr in mixture was substantially (*t*-test: df = 4, *t =* 5.34, *p* = 0.0059) reduced when compared with that of the solo Mr cells (Figure 2A,C). We also tested fungal penetration of cellophane membranes. Three days post inoculation, we found that Mr but not Bb could penetrate the membrane, and the inoculation of the Bb/Mr spore mixture (1:1) failed in penetrating the membrane (Figure 2D). Thus, an antagonistic effect was evident between fungi from the start of spore germination and differentiation.

### 3.5. One-Sided Dominance of M. robertsii within Insect Hemocoels

**Figure 3:**
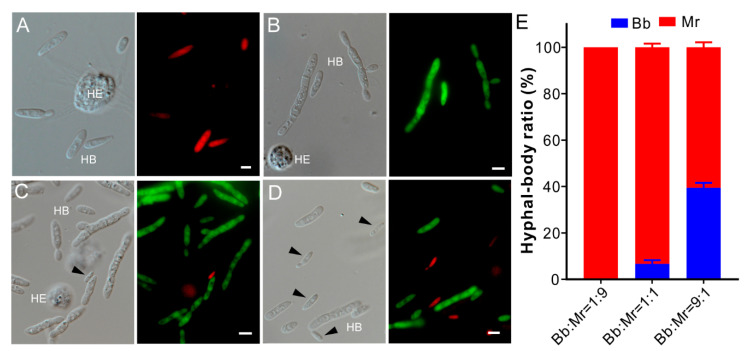
Parasite competition within insect hemocoels. (**A**) Hyphal body (HB) cells of the RFP-labelled Bb formed in insects. (**B**) HB cells of the GFP-labelled Mr formed in insects. (**C**) HB cells formed in insects of both fungi injected with the suspension mixture at the Bb/Mr ratio of 1:1. (**D**) HB cells formed in insects of both fungi injected with the suspension mixture at the Bb/Mr ratio of 9:1. The insects were bled for imaging 60 h post injection. For each panel, the left ones show the bright-field image and the right ones show the corresponding fluorescence image. HE, insect hemocyte; Bb cells are arrowed in panels (**C**,**D**). Bar, 5 µm. (**E**) Ratio estimation of the HB cells formed within insects after injection with spore mixtures for 60 h. Strains: Bb, *Beauveria bassiana* ARSEF 2860; Mr, *Metarhizium robertsii* ARSEF 23.

Next, we tested fungal competition within insect body cavities by injection of the wax moth larvae with the RFP or GFP-labelled Bb and Mr spores or their mixtures. The same time (60 h) post injection, a large amount of the hyphal body (HB) cells could be produced by either Bb or Mr alone within insect hemocoels (Figure 3A,B). For the co-injections, the HB cells of RFP-Bb could be hardly detected in insect hemolymph for the treatment of the Bb/Mr mixture at a 1:9 ratio, i.e., being fully occupied by Mr cells. For the co-injection of the Bb/Mr mixtures at either 1:1 (*t*-test: df = 2, *t =* 55.67, *p =* 0.0003) or 9:1 (*t*-test: df = 2, *t =* 9.89, *p =* 0.01) ratios, significantly fewer RFP-Bb cells could be observed when compared with the GFP-Mr cells in insects (Figure 3C–E). Thus, Mr could markedly outcompete Bb within the insect body cavity.

### 3.6. Dominance of B. bassiana during Co-Culturing in Artificial Medium

**Figure 4:**
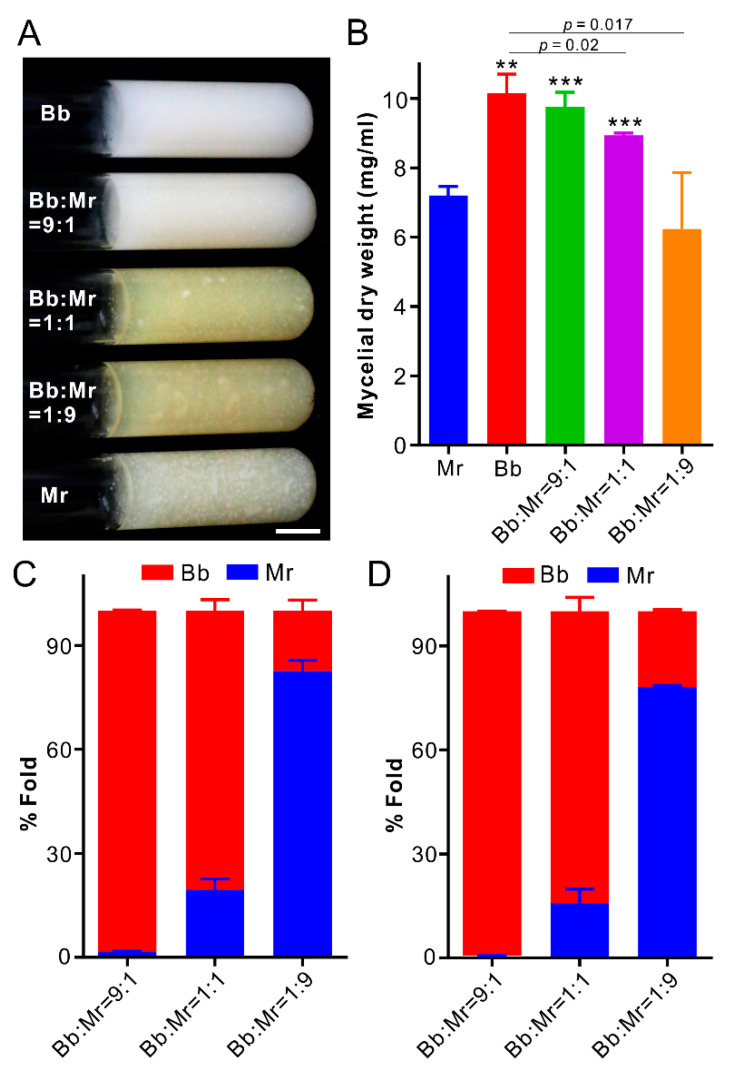
Parasite competition in liquid medium. (**A**) Phenotype of fungal cultures. Fungal spores or spore mixtures were inoculated in SDB for seven days. Bar, 1 cm. (**B**) Comparison of mycelial biomasses. The significance of the difference was tested against either Mr or Bb. The asterisks above columns showing the comparison with Mr at the difference level: ** *p* < 0.01; *** *p* < 0.001. The *p* values showing above the columns indicate the difference levels between Bb and relative mixture treatments, i.e., *p =* 0.02 between Bb and Bb:Mr = 1:1; *p =* 0.017 between Bb and Bb:Mr = 1:9. Quantification analysis of fungal DNA ratio in co-cultures three (**C**) and seven (**D**) days post inoculation. Strains: Bb, *Beauveria bassiana* ARSEF 2860; Mr, *Metarhizium robertsii* ARSEF 23.

Seven days post co-culturing in SDB (Figure 4A), Bb mycelial biomass was significantly higher (*t*-test: df = 4, *t* = 8.31, *p* = 0.0014) than that of Mr, indicating that Bb grew faster than Mr in liquid medium. The biomasses of the co-cultures Bb/Mr at the 9:1 and 1:1 ratios were significantly higher (*t*-test: df = 4, *t* > 9, *p* < 0.001) than that of solo Mr, whereas the Bb/Mr 1:1 and 1:9 biomasses were considerably lower (*t*-test: df = 4, *t* > 3, *p* < 0.05) than that of solo Bb (Figure 4B). We also performed DNA quantification analysis, and the results indicated that Bb could outcompete Mr from an equal start. At the 1:1 inoculation ratio, Mr only took 19.5 ± 3.2% of the co-cultures three days post inoculation, but 15.8 ± 4.1% of the co-cultures seven days post inoculation (Figure 4C,D). At the Bb/Mr ratio of 9:1, Mr could be almost completely wiped off after co-culturing for seven days (Figure 4D). After co-inoculation of the RFP-Bb and GFP-Mr spores in SDB at the 1:1 ratio, microscopic examination indicated that Bb but not Mr started to produce blastospores 24 h post inoculation, and a large amount of these propagules could be produced by Bb after inoculation for 48 h (Figure S4). This kind of strategy might be helpful for Bb to quickly outcompete Mr during co-growth in artificial medium.

### 3.7. Biased Mycosis of Insects after Infection with Different Strains

Next, we were curious to know the outcome of competitions between strains. For two Mr strains, ARSEF 23 grew faster than ARSEF 2575 (*t*-test: df =4; *t* = 6.41, *p =* 0.003 for the cultures 11 day post inoculation) (Figure S1B), and the Bb strain ARSEF 8028 grew faster than ARSEF 2860 on PDA (*t*-test: df =4, *t =* 5.88, *p =* 0.0042 for the cultures 11 day post inoculation) (Figure S1C). The non-synergistic effect on virulence was similarly evident in the experiments of both topical infection and infection assays (Figure S5; Tables S5–S8). For example, the *Metarhizium* strain ARSEF 23 could more quickly (log-rank test: df = 1, χ^2^ = 50.01, *p* < 0.0001) kill insects than ARSEF 2575 during topical infection of the wax moth larvae, and the insect survival curves of their mixtures were in between the dynamics of the two mother strains. Insect survivals were similar (log-rank test: df = 1, χ^2^ = 0.17, *p* = 0.682) after topical infection of two *Beauveria* strains, and the non-significant relationship of insect survival was generally observed between each mother strain and their mixtures.

**Figure 5:**
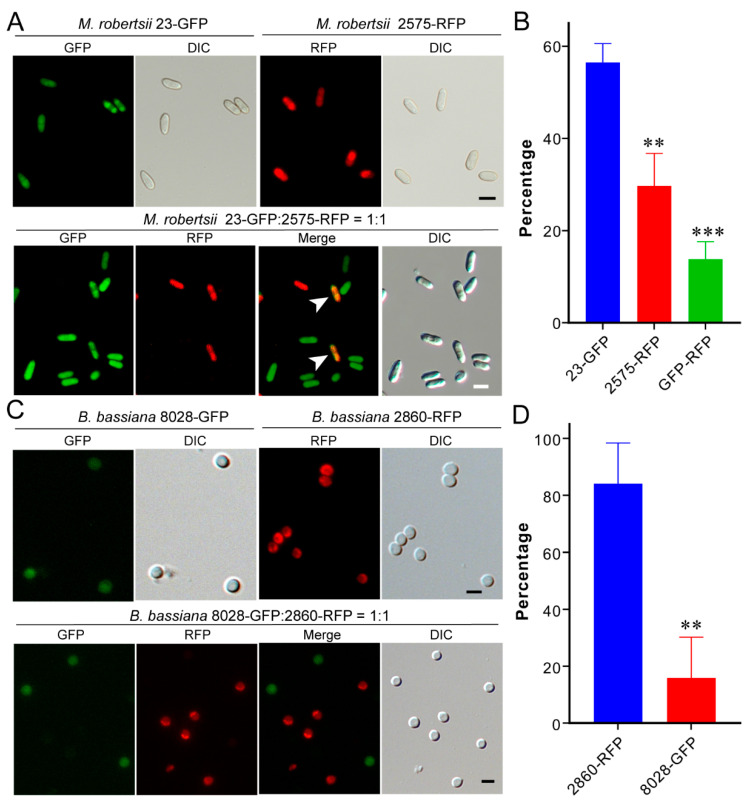
Competition between fungal strains in mycosis of insect cadavers. (**A**) Fluorescent conidia produced by two *Metarhizium* strains on insect cadavers. Conidial spores containing both GFP and RFP signals were arrowed. Bar, 5 µm. (**B**) Percentage of the *Metarhizium* spores with different types of fluorescent signals. (**C**) Fluorescent conidia produced by two *Beauveria* strains on insect cadavers. Bar, 5 µm. (**D**) Percentage of the *Beauveria* spores with different types of fluorescent signals. The asterisks above the columns of the panels B and D show the difference levels between strains at ** *p* < 0.01; *** *p* < 0.001.

Considering that the cadavers are indistinguishable after mycosis by different strains of either *Metarhizium* or *Beauveria*, the fluorescent protein-labelled strains were generated and used for mycosis assays. Thus, the spores of the obtained 23-GFP and 2575-RFP of *M. robertsii*, and the 2860-RFP and 8028-GFP of *B. bassiana* were mixed (1:1), respectively, to co-inject the wax moth larvae. After insect mycosis, we found that the fluorescent spores were unequally produced on insects. For two *Metarhizium* strains, 23-GFP spores were largely (56.5 ± 4.1%) produced on cadavers over 2575-RFP spores (29.7 ± 7.1%; *t*-test: df = 8, *t =* 7.36, *p* = 0.005). It was interesting to find a proportion of spores (13.8 ± 3.8%) containing both the GFP and RFP signals (Figure 5A,B), an indication of parasexual recombination. For the coinfections with two *Beauveria* strains, it was found that 2860-RFP (84.1 ± 14.3%) dominated the mycosis of insect cadavers over 8028-GFP (15.9 ± 14.1%; *t*-test: df = 8, *t =* 5.85, *p* = 0.004) (Figure 5C,D). In contrast to the *Metarhizium* strains, these two *Beauveria* strains did not produce progenies containing the signals of both fluorescent proteins after co-infection of insects.

## 4. Discussion

It is common that mammal or plant individuals are infected at the same time by multiple parasites [33,34,35,36]. Different models or theories have been proposed; however, empirical supports are largely needed. In this study, we performed coinfections of insects with different species or strains of fungal parasites and found that the relatively small-sized insect individuals were killed and largely mycosed by a single species/strain. Consistently, previous coinfection of insects also resulted in the dominant mycosis of insects by single species of parasitic fungi [18,37,38]. Taken together, our empirical data suggest that the competitive exclusion principle (CEP) would fit for the pattern and outcome of fungal parasite competitions for insect individuals. Regarding the two examined species *B. bassiana* and *M. robertsii*, the latter would be a superior competitor since it could grow quicker and kill insects faster than *B. bassiana*. In addition, we found that the dominant mycosis of insect cadavers by *M. robertsii* was largely independent of its initial dosage titer in coinfection and the order of inoculation. Considering the diversity of competing species, CEP postulates that competitors can coexist if they occupy distinct ecological niches [12,39]. Indeed, the diverse species and strains of fungal insect parasites apparently co-occur in different ecosystems [10,40,41]. We also found that the “inferior” competitor *B. bassiana* could outcompete *M. robertsii* when two fungi were co-cultured in an artificial medium, a condition mimicking fungal saprophytic propagation in the field. Our findings thus not only support the fit for CEP between the competitions of fungal insect parasites but also unveil the alternative strategy employed by the inferior competitors to maintain their persistence in distinct niches. Except for the finding that *M. robertsii* grows more quickly than *B. bassiana*, an additional mechanism or mechanisms remains to be determined why the former can outcompete *Beauveria* in coinfections.

Ascomycete insect pathogens are closely related to plant pathogens and endophytes [42]. The DNA barcoding analysis of wood endophytes revealed that CEP was not applicable to these fungi since diverse fungal taxa were co-present in wood samples [43]. Instead, the model of competition–colonization tradeoffs has been suggested to mediate the competition and coexistence of the fungal mycorrhiza or pathogens since the diverse species of these fungi could either occupy different root tips [13,44,45] or infect different parts of individual plants [34,36]. We found that the infection order of *M. robertsii* barely affected its dominant mycosis ability, i.e., the unidirectional competition without the obvious priority effect. It has also been found that the filed applications of the *B. bassiana* and *M. robertsii* strains for pest controls could persist in local populations for the long term [7,46]. Taken together, the modern coexistence theory, distinguished as a priority effect [47,48], might also be inapplicable to the competition between insect parasites. Considering the high density, multispecies concurrence, and small-sized features of insects, the CEP patterns would therefore be common between the competitions of insect parasites. In contrast to the generalist *B. bassiana* and *M. robertsii*, each with broad host ranges, the entomopathogens such as the acridid-specific pathogen *Metarhizium acridum* and the zombie-ant fungi have narrow host ranges [24,49]. Further investigations are still required to determine the competition and coexistence patterns between those specialist pathogens as well as between fungal and non-fungal parasites.

The phrase within-host competition has been frequently used in theoretical ecologies. Actually, both animal and plant parasites/endophytes infect hosts through a multistep process of colonization. We examined the competition between two fungal parasites at both the outside and inside insect levels and found the competition actually occurred at both stages. At least due to the nutrient limitation on insect surfaces, it is not surprising to find that the dual inhibition of spore germination (*B. bassiana*) or appressorium differentiation (*M. robertsii*) occurred at the initial stage of fungal infection. At this stage, it could be concluded that *M. robertsii* outcompeted *B. bassiana* from the spore-germination point of view. The direct injection assays also evidenced that *M. robertsii* cells dominated insect hemocoels, which led to the exclusive mycosis of insect cadavers by this fungus. Thus, competitive exclusion occurred at different steps, probably at every step, between parasites during the infection and mycosis of insects. This kind of the dynamic dual inhibition and exclusive competition could explain why the synergistic effect was not observed in coinfections in this study and as being reported [50]. In this respect, the mixed use of fungal parasites for pest control is not recommended.

In addition to the wax moth larvae, our additional coinfections of fruit flies and mealworms revealed that *M. robertsii* dominated the mycosis of flies, but a much high ratio of co-mycosis occurred during the coinfection of mealworms. The results suggest that the divergent species of insect hosts might affect the outcomes of parasite competition. The host immune modulation of parasite competition has been proposed in parasite–host interactive systems [51,52]. At least, for example, different numbers and families of antimicrobial peptide genes are encoded by different insects [53]. It was intriguing to find that the coinfection of ants or termites with *M. anisopliae* and the facultative saprophyte *Aspergillus* species largely resulted in the mycosis of insect cadavers by *Aspergillus*. It was assumed that host immunity was first inhibited by *Metarhizium*, which led to the mycosis of insects by the fast-growing saprophytes [54,55]. The coinfection of ants with different *Metarhizium* strains and species (i.e., *M. robertsii* and *M. brunneum*) revealed that insect social immunity (sanitary care by grooming to reduce spore load) also affect the consequence of parasite competitions [56]. Thus, the host effect on competition can be species-dependent, which requires further investigation.

During co-culturing in artificial medium, we found that, in contrast to *M. robertsii*, *B. bassiana* evolved with the ability to produce blastospores shortly after inoculation that might enable the fungus to outcompete *Metarhizium* species. Thus, both the host and non-host patch traits may influence the outcomes of parasite competition and coexistence. In addition, different strains and species of fungal parasites also demonstrate the feature of host preference, manifested in the varied speed of insect killing [7,57,58]. Otherwise, the environmental factors such as temperature and ultraviolet radiation also affect the outcomes of competition and coexistence of fungal parasites [19,20,50]. Thus, along with the pattern of exclusive competition for individual insects, fungal insect parasites evolved with multiple ways/abilities to coexist in distinct ecological niches. It has been confirmed in recent years that the ascomycete insect pathogens such as *Metarhizium* and *Beauveria* species can form endophytic or rhizospheric relationships with plants for trading nutrients [59,60]. Obviously, it is the additional strategy for these fungi to maintain persistence or coexistence in the field, especially in the periodic seasons without insect hosts. The pattern of their competition for plants can be similar to other fungal endophytes or mycorrhiza but remains to be determined in the future.

It is mixed regarding the effect of species relatedness on their coexistence and competition, since the more closely related species can be either more or less likely to coexist [61]. The study of animals indicated that the inter-specific rivalry is usually greater than intra-specific competition [62]. We found that, unlike the CEP pattern of interspecies competition, intra-species coinfections resulted in the production of both genotypic spores on cadavers, even with a unidirectional bias towards one strain of either *M. robertsii* or *B. bassiana*. The sexual cycle of the heterothallic *Metarhizium* and *Beauveria* species rarely occurs in the field [63]. Instead, parasexual recombination takes place between compatible strains through the process of hyphal fusion, heterokaryosis, and mitotic crossing-over for limited genetic recombination [7,63]. We found a decent ratio of the *Metarhizium* spores containing both the GFP and RFP signals, good support for recombination between two examined strains. However, the case was not evident between two *Beauveria* strains, which might be due to the issue of heterokaryon incompatibility that can exclude competing kinships to limit the spread of harmful nuclear or cytoplasmic elements [64]. Thus, it seems that exclusive competition may occur between incompatible strains but recombination arises from the compatible strains of fungal insect parasites.

In conclusion, our empirical data support the principle of competitive exclusion fitting for the fungal parasite infection and mycosis of insect individuals at both the inter- and intra-species levels. Different species or strains of parasites can coexist sympatrically by occupying distinct ecological niches including the alternative infection of the individual insects belonging to the same or different species. In addition to filling this knowledge gap, the findings of study are also of practical importance, namely that care has to be taken before considering the mixed use of fungal parasites for insect pest control.

## Data Availability

Not applicable.

## Supplementary Materials

The following are available online at https://www.mdpi.com/article/10.3390/jof7050385/s1, Figure S1: Pairing growth of fungi on PDA for different times; Figure S2: Insect survivals; Figure S3: Biased mycosis; Figure S4: Microscopic examination of the time-scale growth of the co-inoculated RFP-Bb and GFP-Mr (1:1) in SDB for different time; Figure S5: Survival of the wax moth larvae after infections with divergent fungal strains. Table S1: Statistical comparison of the median lethal time (LT_50_) between Bb and Mr after the topical infection assays against the last instar wax moth larvae; Table S2: Statistical comparison of the median lethal time (LT_50_) between Bb and Mr after the injection assays against the last instar wax moth larvae; Table S3: Statistical comparison of the median lethal time (LT_50_) between Bb and Mr after the topical infection assays against the female adults of fruit flies; Table S4: Statistical comparison of the median lethal time (LT_50_) between Bb and Mr after the topical infection assays against the last instar mealworm larvae; Table S5: Statistical comparison of the median lethal time (LT_50_) between two strains of *M. robertsii* after the topical infection assays against the last instar wax moth larvae; Statistical comparison of the median lethal time (LT_50_) between two strains of *M. robertsii* after the injection assays against the last instar wax moth larvae; Table S7: Statistical comparison of the median lethal time (LT_50_) between two strains of *B. bassiana* after the topical infection assays against the last instar wax moth larvae; Statistical comparison of the median lethal time (LT_50_) between two strains of *M. robertsii* after the injection assays against the last instar wax moth larvae.

## References

[1] Stork N.E. (2018). How many species of insects and other terrestrial arthropods are there on earth?. Annu. Rev. Entomol..

[2] Mora C., Tittensor D.P., Adl S., Simpson A.G., Worm B. (2011). How many species are there on Earth and in the ocean?. PLoS Biol..

[3] Lovett B., St Leger R.J. (2017). The insect pathogens. Microbiol. Spectr..

[4] St Leger R.J., Wang J.B. (2020). *Metarhizium*: Jack of all trades, master of many. Open Biol..

[5] Steinwender B.M., Enkerli J., Widmer F., Eilenberg J., Kristensen H.L., Bidochka M.J., Meyling N.V. (2015). Root isolations of *Metarhizium* spp. from crops reflect diversity in the soil and indicate no plant specificity. J. Invertebr. Pathol..

[6] Meyling N.V., Eilenberg J. (2007). Ecology of the entomopathogenic fungi *Beauveria bassiana* and *Metarhizium anisopliae* in temperate agroecosystems: Potential for conservation biological control. Biol. Control.

[7] Mei L.J., Chen M., Shang Y., Tang G., Tao Y., Zeng L., Huang B., Li Z., Zhan S., Wang C.S. (2020). Population genomics and evolution of a fungal pathogen after releasing exotic strains to control insect pests for 20 years. ISME J..

[8] Hajek A.E., St Leger R.J. (1994). Interactions between Fungal Pathogens and Insect Hosts. Annu. Rev. Entomol..

[9] Wang C., Shah F.A., Patel N., Li Z.Z., Butt T.M. (2003). Molecular investigation on strain genetic relatedness and population structure of *Beauveria bassiana*. Environ. Microbiol..

[10] Clifton E.H., Castrillo L.A., Gryganskyi A., Hajek A.E. (2019). A pair of native fungal pathogens drives decline of a new invasive herbivore. Proc. Natl. Acad. Sci. USA.

[11] Bremermann H.J., Thieme H.R. (1989). A competitive exclusion principle for pathogen virulence. J. Math. Biol..

[12] Zheng X.D., Deng L.L., Qiang W.Y., Cressman R., Tao Y. (2017). Limiting similarity of competitive species and demographic stochasticity. Phys. Rev. E.

[13] Smith G.R., Steidinger B.S., Bruns T.D., Peay K.G. (2018). Competition-colonization tradeoffs structure fungal diversity. ISME J..

[14] Harbison C.W., Bush S.E., Malenke J.R., Clayton D.H. (2008). Comparative transmission dynamics of competing parasite species. Ecology.

[15] Chesson P. (2000). Mechanisms of maintenance of species diversity. Annu. Rev. Ecol. Syst..

[16] Wang C.S., Feng M.G. (2014). Advances in fundamental and applied studies in China of fungal biocontrol agents for use against arthropod pests. Biol. Control.

[17] de Faria M.R., Wraight S.P. (2007). Mycoinsecticides and mycoacaricides: A comprehensive list with worldwide coverage and international classification of formulation types. Biol. Control.

[18] Uma Maheswara Rao C., Uma Devi K., Akbar Ali Khan P. (2007). Effect of combination treatment with entomopathogenic fungi *Beauveria bassiana* and *Nomuraea rileyi* (Hypocreales) on *Spodoptera litura* (Lepidoptera: Noctuidaeae). Biocontrol Sci. Technol..

[19] Seid A.M., Fredensborg B.L., Steinwender B.M., Meyling N.V. (2019). Temperature-dependent germination, growth and co-infection of *Beauveria* spp. isolates from different climatic regions. Biocontrol Sci Techn..

[20] Inglis G.D., Johnson D.L., Cheng K.J., Goettel M.S. (1997). Use of pathogen combinations to overcome the constraints of temperature on entomopathogenic hyphomycetes against grasshoppers. Biol. Control.

[21] Wang C., Fan M., Li Z.Z., Butt T.M. (2004). Molecular monitoring and evaluation of the application of the insect-pathogenic fungus *Beauveria bassiana* in southeast China. J. Appl. Microbiol..

[22] Wang C.S., Wang S.B. (2017). Insect pathogenic fungi: Genomics, molecular interactions, and genetic improvements. Annu. Rev. Entomol..

[23] Valero-Jimenez C.A., Faino L., Spring In’t Veld D., Smit S., Zwaan B.J., van Kan J.A. (2016). Comparative genomics of *Beauveria bassiana*: Uncovering signatures of virulence against mosquitoes. BMC Genom..

[24] Hu X., Xiao G., Zheng P., Shang Y., Su Y., Zhang X., Liu X., Zhan S., St Leger R.J., Wang C. (2014). Trajectory and genomic determinants of fungal-pathogen speciation and host adaptation. Proc. Natl. Acad. Sci. USA.

[25] Cen K., Li B., Lu Y.Z., Zhang S.W., Wang C.S. (2017). Divergent LysM effectors contribute to the virulence of *Beauveria bassiana* by evasion of insect immune defenses. PLoS Pathog..

[26] Fang W., Pei Y., Bidochka M.J. (2006). Transformation of *Metarhizium anisopliae* mediated by *Agrobacterium tumefaciens*. Can. J. Microbiol..

[27] Gottar M., Gobert V., Matskevich A., Reichhart J., Wang C., Butt T., BeIvin M., Hoffmann J., Ferrandon D. (2006). Dual detection of fungal infections in *Drosophila* via recognition of glucans and sensing of virulence factors. Cell.

[28] Tang G.R., Shang Y.F., Li S.Q., Wang C.S. (2020). MrHex1 is required for Woronin body formation, fungal development and virulence in *Metarhizium robertsii*. J. Fungi.

[29] Huang A., Lu M., Ling E., Li P., Wang C.S. (2020). A M35 family metalloprotease is required for fungal virulence against insects by inactivating host prophenoloxidases and beyond. Virulence.

[30] Shang J.M., Shang Y.F., Tang G.R., Wang C.S. (2021). Identification of a key G-protein coupled receptor in mediating appressorium formation and fungal virulence against insects. Sci. China Life Sci..

[31] Chen Y.X., Li B., Cen K., Lu Y.Z., Zhang S.W., Wang C.S. (2018). Diverse effect of phosphatidylcholine biosynthetic genes on phospholipid homeostasis, cell autophagy and fungal developments in *Metarhizium robertsii*. Environ. Microbiol..

[32] Wang C.S., St Leger R.J. (2007). The *Metarhizium anisopliae* perilipin homolog MPL1 regulates lipid metabolism, appressorial turgor pressure, and virulence. J. Biol. Chem..

[33] Alizon S., de Roode J.C., Michalakis Y. (2013). Multiple infections and the evolution of virulence. Ecol. Lett..

[34] López-Villavicencio M., Jonot O., Coantic A., Hood M.E., Enjalbert J., Giraud T. (2007). Multiple infections by the anther smut pathogen are frequent and involve related strains. PLoS Pathog..

[35] Susi H., Barres B., Vale P.F., Laine A.L. (2015). Co-infection alters population dynamics of infectious disease. Nat. Commun..

[36] Abbate J.L., Gladieux P., Hood M.E., de Vienne D.M., Antonovics J., Snirc A., Giraud T. (2018). Co-occurrence among three divergent plant-castrating fungi in the same Silene host species. Mol. Ecol..

[37] Pauli G., Moura Mascarin G., Eilenberg J., Delalibera Junior I. (2018). Within-host competition between two entomopathogenic fungi and a granulovirus in *Diatraea saccharalis* (Lepidoptera: Crambidae). Insects.

[38] Wang C., Li Z., Butt T. (2002). Molecular studies of co-formulated strains of the entomopathogenic fungus, *Beauveria bassiana*. J. Invertebr. Pathol..

[39] Adler P.B., Fajardo A., Kleinhesselink A.R., Kraft N.J. (2013). Trait-based tests of coexistence mechanisms. Ecol. Lett..

[40] Meyling N.V., Lubeck M., Buckley E.P., Eilenberg J., Rehner S.A. (2009). Community composition, host range and genetic structure of the fungal entomopathogen *Beauveria* in adjoining agricultural and seminatural habitats. Mol. Ecol..

[41] Ramos Y., Portal O., Lysoe E., Meyling N.V., Klingen I. (2017). Diversity and abundance of *Beauveria bassiana* in soils, stink bugs and plant tissues of common bean from organic and conventional fields. J. Invertebr. Pathol..

[42] Shang Y.F., Xiao G.H., Zheng P., Cen K., Zhan S., Wang C.S. (2016). Divergent and convergent evolution of fungal pathogenicity. Genome Biol. Evol..

[43] Lee M.R., Powell J.R., Oberle B., Cornwell W.K., Lyons M., Rigg J.L., Zanne A.E. (2019). Good neighbors aplenty: Fungal endophytes rarely exhibit competitive exclusion patterns across a span of woody habitats. Ecology.

[44] Kennedy P. (2010). Ectomycorrhizal fungi and interspecific competition: Species interactions, community structure, coexistence mechanisms, and future research directions. New Phytol..

[45] Bennett A.E., Bever J.D. (2009). Trade-offs between arbuscular mycorrhizal fungal competitive ability and host growth promotion in *Plantago lanceolata*. Oecologia.

[46] Wang S., O’Brien T.R., Pava-Ripoll M., St Leger R.J. (2011). Local adaptation of an introduced transgenic insect fungal pathogen due to new beneficial mutations. Proc. Natl. Acad. Sci. USA.

[47] Grainger T.N., Letten A.D., Gilbert B., Fukami T. (2019). Applying modern coexistence theory to priority effects. Proc. Natl. Acad. Sci. USA.

[48] Levine J.M., HilleRisLambers J. (2009). The importance of niches for the maintenance of species diversity. Nature.

[49] Araujo J.P.M., Evans H.C., Kepler R., Hughes D.P. (2018). Zombie-ant fungi across continents: 15 new species and new combinations within *Ophiocordyceps*. I. Myrmecophilous hirsutelloid species. Stud. Mycol..

[50] Thomas M.B., Watson E.L., Valverde-Garcia P. (2003). Mixed infections and insect-pathogen interactions. Ecol. Lett..

[51] Ulrich Y., Schmid-Hempel P. (2012). Host modulation of parasite competition in multiple infections. Proc. Biol. Sci..

[52] de Roode J.C., Culleton R., Cheesman S.J., Carter R., Read A.F. (2004). Host heterogeneity is a determinant of competitive exclusion or coexistence in genetically diverse malaria infections. Proc. Biol. Sci..

[53] Duwadi D., Shrestha A., Yilma B., Kozlovski I., Sa-Eed M., Dahal N., Jukosky J. (2018). Identification and screening of potent antimicrobial peptides in arthropod genomes. Peptides.

[54] Hughes W.O., Boomsma J.J. (2004). Let your enemy do the work: Within-host interactions between two fungal parasites of leaf-cutting ants. Proc. Biol. Sci..

[55] Chouvenc T., Efstathion C.A., Elliott M.L., Su N.-Y. (2012). Resource competition between two fungal parasites in subterranean termites. Naturwissenschaften.

[56] Milutinovic B., Stock M., Grasse A.V., Naderlinger E., Hilbe C., Cremer S. (2020). Social immunity modulates competition between coinfecting pathogens. Ecol. Lett..

[57] Maistrou S., Natsopoulou M.E., Jensen A.B., Meyling N.V. (2020). Virulence traits within a community of the fungal entomopathogen *Beauveria*: Associations with abundance and distribution. Fungal Ecol..

[58] Klinger E.G., Vojvodic S., DeGrandi-Hoffman G., Welker D.L., James R.R. (2015). Mixed infections reveal virulence differences between host-specific bee pathogens. J. Invertebr. Pathol..

[59] Behie S.W., Zelisko P.M., Bidochka M.J. (2012). Endophytic insect-parasitic fungi translocate nitrogen directly from insects to plants. Science.

[60] Behie S.W., Moreira C.C., Sementchoukova I., Barelli L., Zelisko P.M., Bidochka M.J. (2017). Carbon translocation from a plant to an insect-pathogenic endophytic fungus. Nat. Commun..

[61] Mayfield M.M., Levine J.M. (2010). Opposing effects of competitive exclusion on the phylogenetic structure of communities. Ecol. Lett..

[62] Potts J.R., Petrovskii S.V. (2017). Fortune favours the brave: Movement responses shape demographic dynamics in strongly competing populations. J. Theor. Biol..

[63] Zheng P., Xia Y., Zhang S., Wang C. (2013). Genetics of *Cordyceps* and related fungi. Appl. Microbiol. Biotechnol..

[64] Saupe S.J. (2000). Molecular genetics of heterokaryon incompatibility in filamentous ascomycetes. Microbiol. Mol. Biol. Rev..

