# Supplementary material for: Empirical Support for the Pattern of Competitive Exclusion between Insect Parasitic Fungi
Source: J Fungi (Basel). 2021 May 14;7(5):385. doi: 10.3390/jof7050385 (PMC8157078; doi:10.3390/jof7050385)
Supplement: Supplementary file 1 [file jof-07-00385-s001.zip › jof-1208395-supplementary.pdf]

Supplementary Figures:

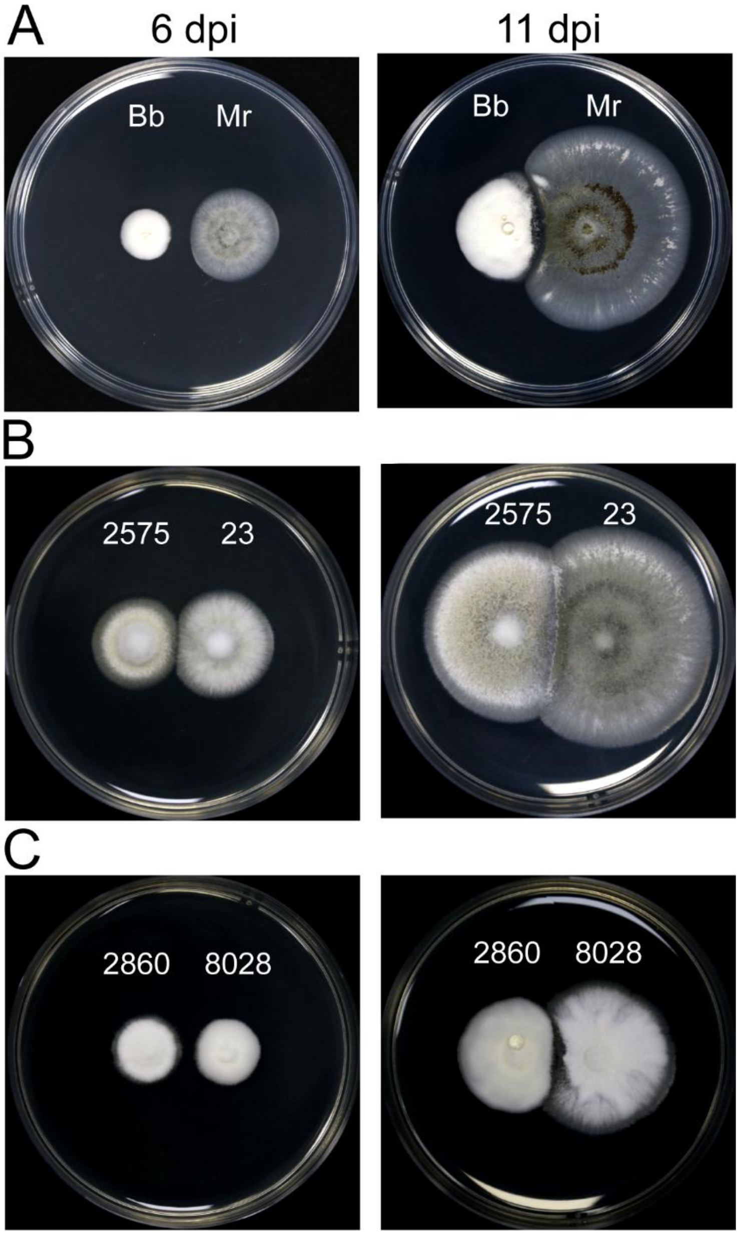

Figure S1 Pairing growth of fungi on PDA for different times. (A) Pairing growth of *B. bassiana* (Bb; ARSEF 2860 strain) and *M. robertsii* (Mr; ARSEF 23 strain). (B) Pairing growth of the two strains of *M. robertsii* (ARSEF 23 and 2575). (C) Pairing growth of the two *B. bassiana* (ASEF 2860 and 8028) on PDA. dpi, days post inoculation.

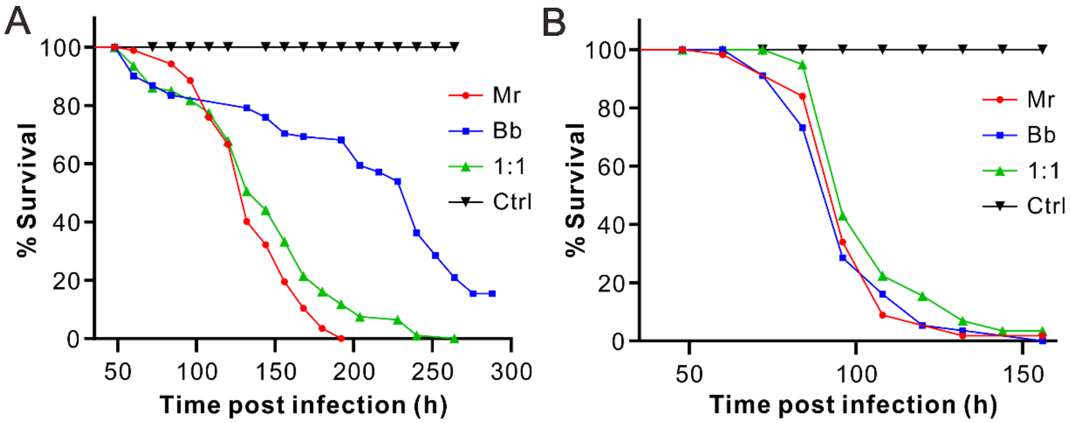

Figure S2 Insect survivals. (A) Survival of the female fruit flies after topical infections. (B) Survival of the mealworm larvae after topical infection. The insects were immersed in the spores suspensions of Bb, Mr and their mixture (1:1) for 30 sec for topical infections. The insects immersed in 0.01% Tween-20 were used after controls

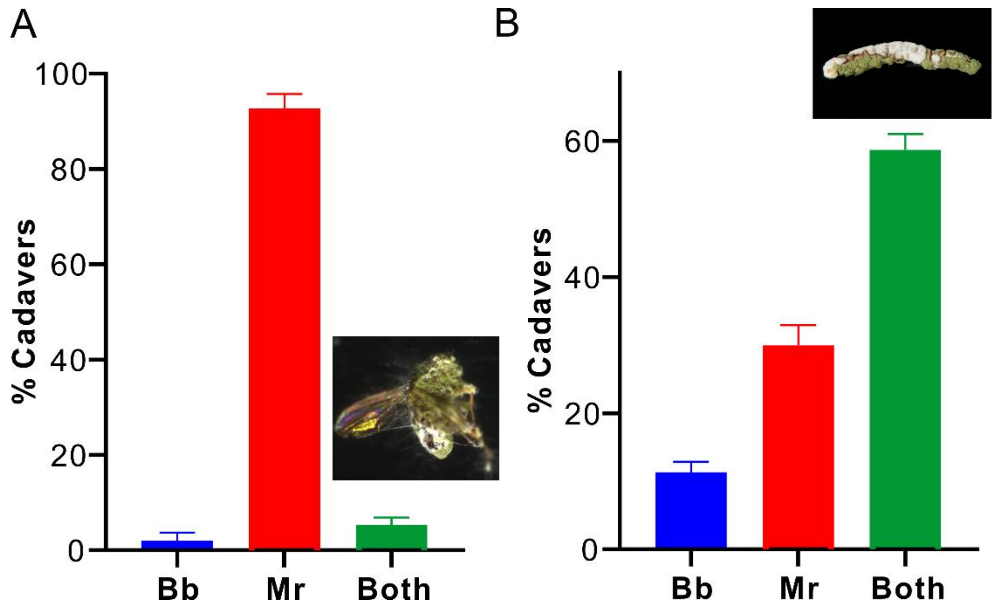

Figure S3 Biased mycosis. Mycosis ratio of the female fruit-fly adults (A) and mealworm larvae (B) after topical infection with the Bb/Mr spore suspension mixture (1:1). Insets showing the insect cadavers co-mycosed by both fungi.

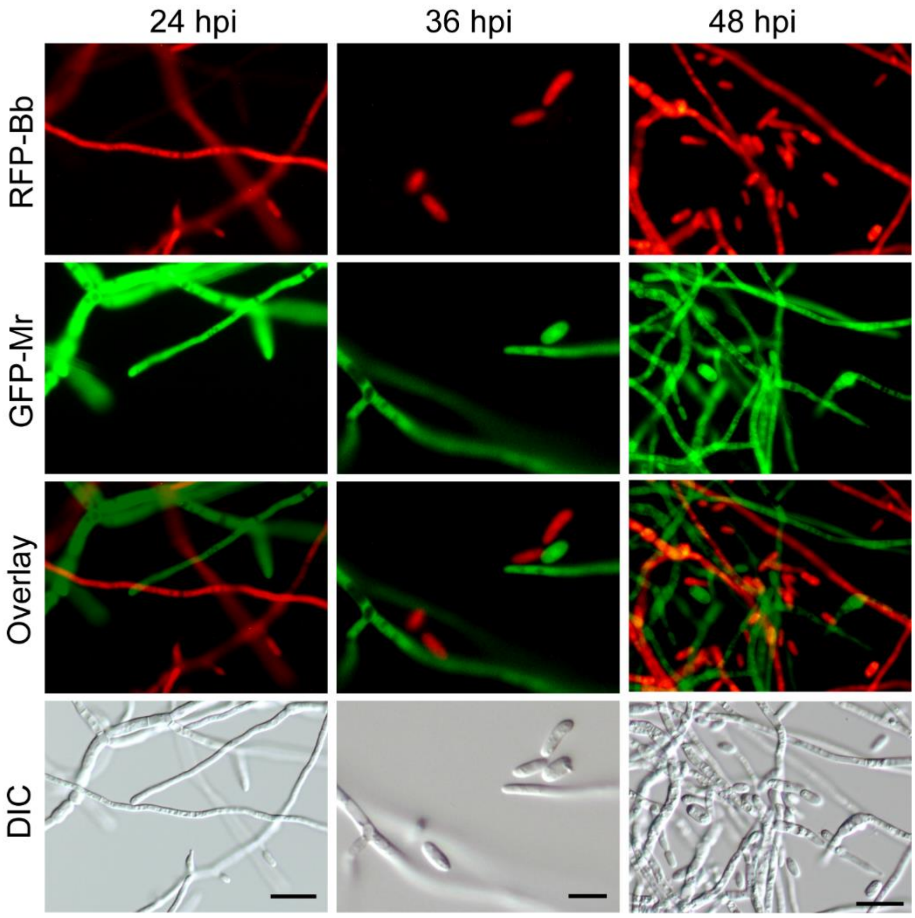

Figure S4 Microscopic examination of the time-scale growth of the co-inoculated RFP-Bb and GFP-Mr (1:1) in SDB for different time. The results showing that the blastospores could be more quickly produced by Bb than Mr. DIC, differential interference contrast. Bar, 5 μm.

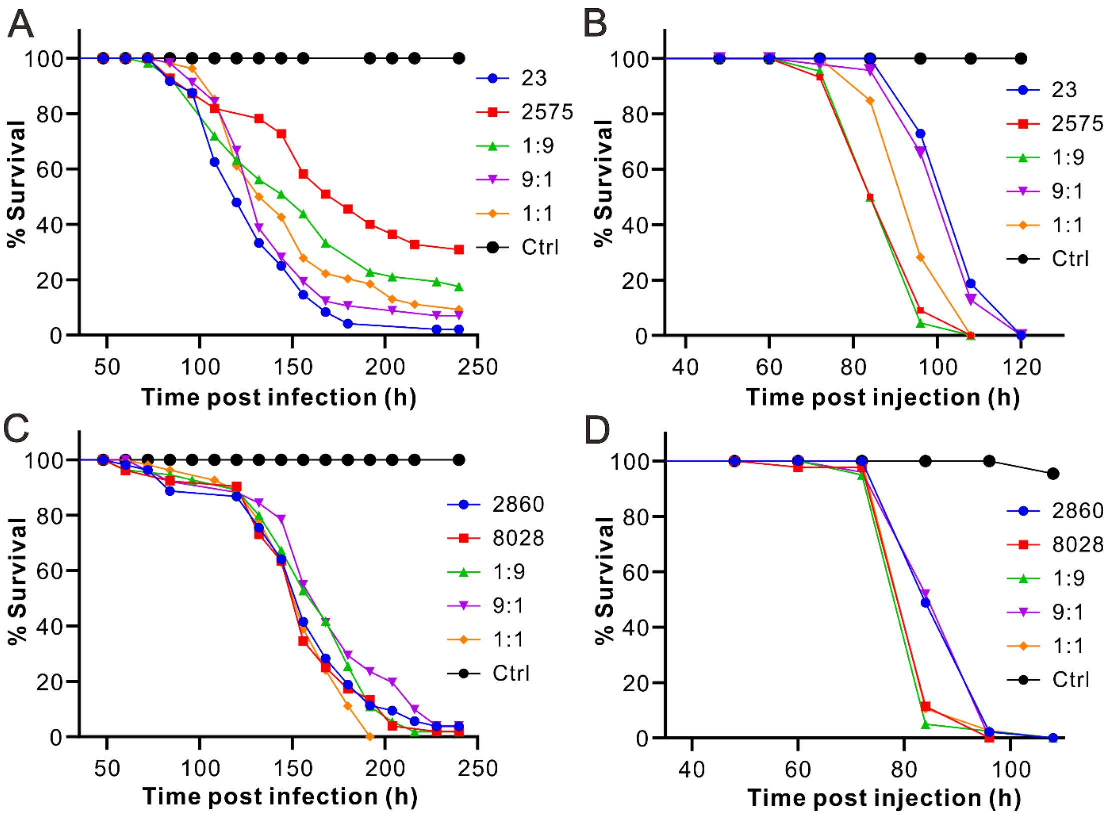

Figure S5 Survival of the wax moth larvae after infections with divergent fungal strains. (A) Survival of the wax moth larvae after topical infection with two *Metarhizium* strains. Spore suspensions (each at 1 × 10^7 conidia/ml) of the individual strain (ARSEF 23 and 2575) and their mixtures (ARSEF 23:2575=1:9; 1:1 and 9:1) were used for immersion assays. (B) Survival of the wax moth larvae after injection with two *Metarhizium* strains. Spore suspensions (each at 1 × 10^6 conidia/ml) of the individual strain and their mixtures were used for injection assays. (C) Survival of the wax moth larvae after topical infection with two *Beauveria* strains. Spore suspensions (each at 1 × 10^7 conidia/ml) of the individual strain (ARSEF 2860 and 8028) and their mixtures (2860:8028=1:9; 1:1 and 9:1) were used for immersion assays. (D) Survival of the wax moth larvae after injection with two *Beauveria* strains. Spore suspensions (each at 1 × 10^6 conidia/ml) of the individual strain and their mixtures were used for injection assays. Control insects were treated with 0.01% Tween-20.

Supplementary Tables:

Table S1. Statistical comparison of the median lethal time (LT_{50}) between Bb and Mr after the topical infection assays against the last instar wax moth larvae.

**Strains /treatments**	**LT_{50} (h)**	**Significance of difference***	
		**Bb**	**Mr**
Bb	144.000±4.007	–	χ^2=10.746; *P*=0.001
Mr	108.000±4.165	χ^2=10.746; *P*=0.001	–
Bb : Mr = 1:9	120.000±4.226	χ^2=8.811; *P*=0.003	χ^2=0.745; *P*=0.604
Bb : Mr = 1:1	144.000±6.117	χ^2=1.032; *P*=0.310	χ^2=11.825; *P*=0.001
Bb : Mr = 9:1	144.000±4.663	χ^2=0.650; *P*=0.420	χ^2=13.400; *P*=0.000

*, Log-Rank test of significance.

Table S2. Statistical comparison of the median lethal time (LT_{50}) between Bb and Mr after the injection assays against the last instar wax moth larvae.

**Strains /treatments**	**LT_{50} (h)**	**Significance of difference***	
		**Bb**	**Mr**
Bb	84.000±0.637	–	χ^2=11.124; *P*=0.001
Mr	72.000±1.261	χ^2=11.124; *P*=0.001	–
Bb : Mr = 1:9	84.000±0.000	χ^2=1.651; *P*=0.199	χ^2=7.153; *P*=0.007
Bb : Mr = 1:1	84.000±0.440	χ^2=0.974; *P*=0.324	χ^2=7.821; *P*=0.005
Bb : Mr = 9:1	84.000±1.514	χ^2=3.446; *P*=0.063	χ^2=20.824; *P*=0.000

*, Log-Rank test of significance.

Table S3. Statistical comparison of the median lethal time (LT_{50}) between Bb and Mr after the topical infection assays against the female adults of fruit flies.

**Strains /treatments**	**LT_{50} (h)**	**Significance of difference***	
		**Bb**	**Mr**
Bb	240.000±5.793	–	χ^2=80.221; *P*=0.000
Mr	132.000±2.386	χ^2=80.221; *P*=0.000	–
Bb : Mr = 1:1	144.000±5.223	χ^2=60.438; *P*=0.000	χ^2=5.990; *P*=0.014

*, Log-Rank test of significance.

Table S4. Statistical comparison of the median lethal time (LT_{50}) between Bb and Mr after the topical infection assays against the last instar mealworm larvae.

**Strains /treatments**	**LT_{50} (h)**	**Significance of difference***	
		**Bb**	**Mr**
Bb	96.000±1.623	–	χ^2=0.614; *P*=0.433
Mr	96.000±1.518	χ^2=0.614; *P*=0.433	–
Bb : Mr = 1:1	96.000±1.509	χ^2=5.742; *P*=0.017	χ^2=3.405; *P*=0.065

*, Log-Rank test of significance.

Table S5. Statistical comparison of the median lethal time (LT_{50}) between two strains of *M. robertsii* after the topical infection assays against the last instar wax moth larvae.

Strains /treatments	LT_{50} (h)	Significance of difference*	
		ARSEF 23	ARSEF 2575
ARSEF 23	120.000±5.933	–	χ^2=29.309; *P*=0.000
ARSEF 2575	180.000±13.294	χ^2=29.309; *P*=0.000	–
ARSEF 23 : 2575 = 1:9	156.000±12.845	χ^2=11.975; *P*=0.001	χ^2=3.788; *P*=0.052
ARSEF 23 : 2575 = 1:1	132.000±8.818	χ^2=6.488; *P*=0.011	χ^2=10.681; *P*=0.001
ARSEF 23 : 2575 = 9:1	132.000±2.757	χ^2=2.387; *P*=0.122	χ^2=18.753; *P*=0.000

*, Log-Rank test of significance.

Table S6. Statistical comparison of the median lethal time (LT_{50}) between two strains of *M. robertsii* after the injection assays against the last instar wax moth larvae.

Strains /treatments	LT_{50} (h)	Significance of difference*	
		ARSEF 23	ARSEF 2575
ARSEF 23	108.000±1.248	–	χ^2=49.420; *P*=0.000
ARSEF 2575	84.000±2.151	χ^2=49.420; *P*=0.000	–
ARSEF 23 : 2575 = 1:9	84.000±1.990	χ^2=55.516; *P*=0.000	χ^2=0.076; *P*=0.783
ARSEF 23 : 2575 = 1:1	96.000±1.409	χ^2=24.850; *P*=0.000	χ^2=13.215; *P*=0.000
ARSEF 23 : 2575 = 9:1	108.000±1.098	χ^2=1.086; *P*=0.297	χ^2=38.881; *P*=0.000

*, Log-Rank test of significance.

Table S7. Statistical comparison of the median lethal time (LT_{50}) between two strains of *B. bassiana* after the topical infection assays against the last instar wax moth larvae.

Strains /treatments	LT_{50} (h)	Significance of difference*	
		ARSEF 2860	ARSEF 8028
ARSEF 2860	156.000±3.587	–	χ^2=0.168; *P*=0.682
ARSEF 8028	156.000±2.745	χ^2=0.168; *P*=0.682	–
ARSEF 2860 : 8028 = 9:1	168.000±4.440	χ^2=2.337; *P*=0.126	χ^2=4.434; *P*=0.035
ARSEF 2860 : 8028 = 1:1	156.000±3.307	χ^2=1.329; *P*=0.249	χ^2=0.925; *P*=0.336
ARSEF 2860 : 8028 = 1:9	168.000±6.271	χ^2=0.244; *P*=0.621	χ^2=0.764; *P*=0.382

*, Log-Rank test of significance.

Table S8. Statistical comparison of the median lethal time (LT_{50}) between two strains of *M. robertsii* after the injection assays against the last instar wax moth larvae.

Strains /treatments	LT_{50} (h)	Significance of difference*	
		ARSEF 2860	ARSEF 8028
ARSEF 2860	84.000±0.660	—	χ2=15.382; *P*=0.000
ARSEF 8028	84.000±1.330	χ^2=15.382; *P*=0.000	—
ARSEF 2860 : 8028 = 9:1	96.000±1.515	χ^2=0.004; *P*=0.947	χ^2=11.925; *P*=0.001
ARSEF 2860 : 8028 = 1:1	84.000±1.531	χ^2=11.418; *P*=0.001	χ^2=0.220; *P*=0.639
ARSEF 2860 : 8028 = 1:9	84.000±1.427	χ^2=26.074; *P*=0.000	χ^2=2.602; *P*=0.107

*, Log-Rank test of significance.
